# New Horizons in the use of routine data for ageing research

**DOI:** 10.1093/ageing/afaa018

**Published:** 2020-02-10

**Authors:** Oliver M Todd, Jennifer K Burton, Richard M Dodds, Joe Hollinghurst, Ronan A Lyons, Terence J Quinn, Anna Schneider, Katherine E Walesby, Chris Wilkinson, Simon Conroy, Chris P Gale, Marlous Hall, Kate Walters, Andrew P Clegg

**Affiliations:** 1 Academic Unit of Elderly Care and Rehabilitation, Bradford Teaching Hospitals NHS Trust, University of Leeds, Bradford, UK; 2 Leeds Institute for Data Analytics, University of Leeds, Leeds, UK; 3 Academic Section of Geriatric Medicine, Institute of Cardiovascular and Medical Sciences, University of Glasgow, Glasgow G4 OSF, UK; 4 AGE Research Group, Translational and Clinical Research Institute, Newcastle University, Newcastle, UK; 5 Health Data Research UK (HDR-UK), Swansea University, Swansea, UK; 6 School of Health & Social Care, Scottish Centre for Administrative Data Research, Edinburgh Napier University, Edinburgh, UK; 7 Alzheimer Scotland Dementia Research Centre, University of Edinburgh, Edinburgh EH8 9JZ, UK; 8 Leeds Institute of Cardiovascular and Metabolic Medicine, University of Leeds, Leeds, UK; 9 Institute of Cellular Medicine, Newcastle University, Newcastle upon Tyne, UK; 10 Department of Health Sciences, University of Leicester, Leicester, UK; 11 Centre for Ageing Population Studies, Department of Primary Care & Population Health, Institute of Epidemiology & Health Care, University College, London, UK

**Keywords:** ageing, big data, data linkage, electronic health records, health informatics, older people

## Abstract

The past three decades have seen a steady increase in the availability of routinely collected health and social care data and the processing power to analyse it. These developments represent a major opportunity for ageing research, especially with the integration of different datasets across traditional boundaries of health and social care, for prognostic research and novel evaluations of interventions with representative populations of older people. However, there are considerable challenges in using routine data at the level of coding, data analysis and in the application of findings to everyday care. New Horizons in applying routine data to investigate novel questions in ageing research require a collaborative approach between clinicians, data scientists, biostatisticians, epidemiologists and trial methodologists. This requires building capacity for the next generation of research leaders in this important area. There is a need to develop consensus code lists and standardised, validated algorithms for common conditions and outcomes that are relevant for older people to maximise the potential of routine data research in this group. Lastly, we must help drive the application of routine data to improve the care of older people, through the development of novel methods for evaluation of interventions using routine data infrastructure. We believe that harnessing routine data can help address knowledge gaps for older people living with multiple conditions and frailty, and design interventions and pathways of care to address the complex health issues we face in caring for older people.

## Key points

- Routine data have potential to improve quality and efficiency of ageing research.
- Applications include prognostic research, clinical trials and service evaluations.
- Routine data record clinical care and may lack what matters most to older people themselves.
- Incentivising codes that have relevance to older people could improve quality of routine data.
- Progress requires multidisciplinary collaboration, capacity building and developing consensus code lists.

## Introduction

Technological advances in data storage and processing have enabled a magnitude of scale in information that has come to define big data as a disruptor of our age [1]. The growth of electronic health records (eHR) worldwide means that data routinely collected as part of health and social care interactions, across a patient’s life course, are now available for research and quality improvement purposes [2]. Models of data linkage have been developed worldwide, across boundaries of primary and secondary care, and across health and social care systems. Supported by this technology, health and social care are becoming more integrated, proactive and personalised. This Horizons review will consider the benefits, challenges and suggested solutions of harnessing routine data for the purposes of ageing research.

## What are the benefits?

Routine data are being generated from a host of different sources which together form ever more detailed ‘digital traces’ of a person’s health [3]. Clinical data are rich in key symptoms, diagnoses, health status measurements (e.g. weight, blood pressure and smoking status), prescribing, investigations and health service use. Using a unique patient identifier, primary care data are increasingly linked to records from an individual’s hospital attendances, social care interactions, census and death records. There is potential for further linkage to genomic data [4], mobile and wearable technology. Making use of routine data in research can reduce research costs and burden on participants and enables the capture of information in large populations and many clinical events. Data are continuously updated, and they may cover long periods of time. Artificial intelligence (AI) and machine learning techniques enable data analysis at greater pace and in larger datasets than was previously possible [5].

People over the age of 85 years, with frailty or dementia, from ethnic minorities, or living in deprived areas tend to be poorly represented in research [6]. Historically, these groups were often excluded by the nature of a study’s design or methods of recruitment [7]. Additionally, many older people may choose not to take part because they feel too unwell, are already overcommitted with hospital appointments, or for social and health literacy reasons. These personal factors may themselves correlate with risk factors for outcomes, and hence participant selection bias can affect the results. Use of routine data based on, for example, general practice-level consent and de-identification of individuals can largely overcome this form of selection bias [8].

Follow-up of patients without needing their active participation means that participant withdrawal is not as big a problem as observed in traditional cohort studies and trials, where loss to follow-up can be significant. The intensity and duration of follow-up provided by routine data would be prohibitively expensive in a traditional observational study or trial based on participant-level data collection. Routine data also offer comprehensive records of prescriptions across a life course. This information is particularly important in ageing research as many medicines are tested in younger, fitter populations, and there is a recognised need for more robust data on the benefits and harms of drugs when their use is then extended to older populations with frailty, multimorbidity and polypharmacy.

Three key areas where the application of routine data has potentially major benefits for research in ageing are as follows.

### Prediction

Analysis of large scale observational data may be used to develop prediction tools to identify levels of future risk of outcomes, and these can inform shared decision making [9, 10]. Real-time updating of clinical records can enable regular re-validation of prediction models according to up-to-date population health and demographics, as undertaken by updated versions of the Q-Risk cardiovascular risk calculator [11]. The clinical utility of predictors or prognostic models developed in routine care can be tested by applying them to live clinical practice data to deliver stratified care interventions for individuals based on their personal risk profile [12], for example, in the prescription of statins on the basis of a person’s cardiovascular Q-risk [13].

### Clinical trials

For older people, whether testing a new complex intervention, device or drug, routine data can help extend participation in clinical trials. In particular, routine data can be used to:

- Demonstrate heterogeneity in practice for particular clinical decisions. For example, variation in prescribing patterns for people with advancing frailty can identify clinical uncertainty to inform the choice of research question a trial will address.
- Allow for a broad range of evaluation designs including: interrupted time series, trials within cohorts, or cluster randomisation where general practices that are contributing to a routinely available dataset are randomised to intervention delivery or control.
- Measure key age-related health indicators at trial recruitment, such as frailty or dementia and compare these baseline characteristics with the wider population-level data, to provide greater confidence in the generalisability of trial findings.
- Provide follow-up of trial endpoints which is less obtrusive by linkage of trials to participant’s eHR data to measure outcomes [14]. This enables extended follow-up periods that are more meaningful in shared decision making.

### Service evaluation

The measurement of outcomes in routine data may be used to indicate performance of clinical services against regional and national benchmarks. These can be used locally to inform the design of Quality Improvement Projects, but also at a regional or national level to inform service redesign or policy changes [15]. In the UK, Global Digital Exemplars are NHS providers recognised for championing the use of digital technologies and information to deliver improvements in quality of care [16, 17]. Learning healthcare systems, that use routine data, are already established in primary care and in single long-term conditions [18]. There is an opportunity to extend these using routine data to involve patients with multimorbidity and those with complex needs, such as frailty, particularly if we can better capture outcomes that are relevant for older people.

## What are the challenges and possible solutions?

**Figure 1:**
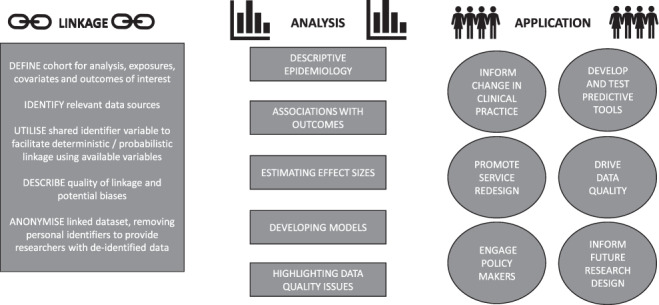
Routine data pathway.

The application of routine data to research questions remains relatively new and there are important methodological challenges involved. We will explore some of the challenges and propose next steps at three stages of the routine data journey: at data linkage, analysis and application (Figure 1).

### Linkage

#### Challenges

Data entry for eHRs is generally undertaken at the time of the clinical or social care interaction, or is subsequently coded from medical notes and correspondence by trained clinical coders. It is helpful to consider both contributors to the data: the individual patient or client who provides the information relevant to the interaction and the practitioner or clinical coder interpreting and entering this information as data.

With respect to the individual, consent for the release of their data collected during the course of normal care tends to follow an opt-out approach, enabling the individual to withdraw their data from databanks used for research or planning purposes [19]. The European Union General Data Protection Regulation requires consent be unambiguous, clearly affirmative, and that clear information is provided on how to withdraw consent for data sharing at any time [20]. A particular challenge of the opt-out model in relation to older people is that there remains debate over whether people who do not have capacity have a realistic option of withdrawing their data.

Routine datasets tend to be pseudonymised, which is defined as personal data that cannot be attributed to an individual without the use of additional information, so long as this additional information is kept separately and that this separation is secure [20]. However, a residual risk of re-identification of individuals from their data typically remains [21]. For example, the relatively low number of centenarians in a particular location could be linked with other freely available information online to identify an individual.

Information governance structures protect the confidentiality of those whose data it is and include the provision of a Data Protection Officer in the University setting or Caldicott guardian in the NHS setting. Applications for data access are reviewed by an independent body in charge of the dataset, according to operating policies directed by the ethics committee when the dataset was originally established. Datasets may also be restricted to remove people with certain rare conditions, and outputs reviewed for risk of potential disclosure by an institution’s data custodians. Researchers involved in the analysis are increasingly required to undertake accredited training to use data appropriately [22].

With respect to the person inputting or coding the data, their primary focus will usually be to record a clinical or non-clinical encounter and the delivery of care, as opposed to recording data for research purposes. One key challenge is that eHRs comprise data from multiple sources with a variety of coding schemes and an event such as a fall may be represented by over 100 different codes in one coding system alone. Multiple factors will have an impact on what is coded and how, introducing recording bias. Coding systems used by healthcare professionals have been updated over time (e.g. Clinical Terms Version 2 or 3 or Systemised Nomenclature of Medicine-Clinical Terms (SNOMED-CT)), and do not always map directly onto one another [23]. Furthermore, the choice of codes available can diverge between computer systems [24] and between prediction models [25]. Coding habits also vary between professionals, clinical practices [26] and over time [17]. Coding may be undertaken for remunerative purposes, particularly in insurance-based healthcare systems, but also relate to incentives and policy change. For example, the Quality Outcomes Framework in England and Wales [27] has significantly influenced the way practitioners record comorbidities [28].

The increasing linkage of shared care records across different data systems are gradually beginning to align common terminologies and standardise their representation [29]. Health Data Research UK is a non-profit organisation working across universities and the NHS to unify UK health data assets across health service, cohort, registry and trial data and facilitate their use in answering key research questions [30].

#### Next steps

Greater advocacy is now needed to improve the representation of and engagement with older people and the conditions of ageing using routine data. There are specific considerations relevant to older people in determining the suitability of routine data to particular research questions and in the need for additional sources of data to capture missing information. Developing a research question that can be answered using routine data is best done in dialogue, and as a collaborative exercise between the disciplines of data analysts, clinicians and social care practitioners.

With this in mind, an Ageing Data Research Collaborative (@geridata) has been formed in the UK with the support of the British Geriatrics Society [31]. This community has been developed to foster peer support among researchers and encourage sharing of pre-analytic protocols, code lists for conditions relevant to geriatric medicine and gerontology, data cleaning and analysis code [32].

### Analysis

#### Challenges

The analyses of routine data require judicious clinical interpretation. The typically large sample sizes of routine datasets can generate estimates with very narrow confidence limits, as the sample estimates converge on the true population mean. This can lead to a situation where results appear statistically significant, but may be clinically unimportant. Therefore, the size of the data does not preclude the need for careful design using the appropriate analyses relevant to the content of the data to minimise bias.

Important information may be missing in routine data. Data may be missing when it should ordinarily be recorded, for example, the date of an event or the dose of a particular medication. Missing data may affect some people more than others because some people will have had fewer opportunities to have their data recorded. On the other hand, there is a tendency for those assessed regularly in routine healthcare, and particularly for those with poor health who are assessed on repeated occasions, to have greater recording of additional diagnoses, sometimes called informed presence bias [33].

Data may also be missing because of the design of routine data records. Routine datasets work on the basis of positive recording: the absence of recording of a particular diagnosis is assumed to represent the absence of diagnosis. Codes tend more often to represent disease simply as present or absent, so that the grade or severity of disease may be difficult to fully account for in routine data analysis [34]. Particular conditions are not routinely recorded in clinical care (e.g. physical disability), or under recorded (e.g. dementia) and the omission or undercounting of such information in a research study could lead to spurious findings [17].

Routine datasets only include data items relevant to the particular clinical or administrative process of care, rather than what is relevant to an individual research question. A loss of higher-order function (cognition, mobility or continence) may be represented by codes for the easiest attributable cause (e.g. urinary tract infection for delirium), whether accurate or not. The use of codes in recording symptoms may be insufficient to represent the complex interaction of multiple pre-disposing and precipitating factors involved in a geriatric syndrome. Where the dominant symptom is the only one recorded, accompanying symptoms will be missing. In these cases, the content of free text may be highly informative, and analysis of narrative text using AI techniques, for example, natural language processing algorithms is an emerging area in ageing research [35].

Transparency of methods is key in validating findings of routine data and this is particularly the case in the development of algorithms using AI and machine learning techniques [5]. Consensus guidelines have been developed specifically for the reporting of routine data studies and recommendations include the publication of a pre-analytic protocol, and the code lists for the variables used alongside any analysis [32].

#### Next steps

We must advocate as a specialty discipline to champion the use of codes to record later-life problems including clinical symptoms experienced in later life, clinical signs of ageing, problems with activities of daily living, impairments and social circumstances alongside more typically recorded diseases. A first step would be to develop consensus code lists for conditions of ageing (i.e. the geriatric giants) mapped across different coding systems. This would improve the efficiency and the standard of research in this area as well as ease comparisons in meta-analyses. Codes characterising key outcomes for older people would then need to be validated using linkage to ageing cohorts. Reaching consensus would require engagement with a wide range of stakeholders, already achieved for cardiovascular outcomes in the validated code lists on the CALIBER UK platform [36]. Consensus could also be reached on what defines likely true and false data recordings (such as the normal reasonable range of physiological metrics, for example, blood pressure, heart rate, body mass index and cholesterol) in the form of data-preparation algorithms.

### Application

#### Challenges

Models built on big data that are co-designed by those with knowledge both of the domain and of the datasets have been shown to improve model performance [37, 38]. However, a major challenge is that the users of routine data research (e.g. health and care professionals, commissioners and policymakers) and its beneficiaries (e.g. older people, their families and carers) have historically not been closely engaged in the research process. Much of data storage and analysis in healthcare still uses the infrastructure and systems developed before the big data era, unintegrated and separate from those involved in the real-life scenarios which routine data applications maybe designed to effect. The incorporation of data-driven techniques to healthcare will be disruptive and a lack of collaborative working could jeopardise progress. If the processes of testing and validation in real-life scenarios require interruption of clinical services, the application of routine data to clinical care will be slow or non-existent [39].

In the UK, in response to the National Data Guardian’s call for better public conversation about health data, an independent initiative was set up called ‘Understanding Patient Data’ funded by the Wellcome Trust [5]. The initiative tries to help make patient data more visible, understandable and trustworthy to the public.

#### Next Steps

An ageing big data research user group, with key representation from older people, carers, professionals, commissioners and policymakers, is needed, with a specific focus on driving implementation of findings into routine practice. Better engagement of users and beneficiaries of routine data research will provide insights that the data alone cannot capture. This broader collaboration could inform the development of research questions, research design and interpretation of findings [40]. In the future, we need co-development of core outcome sets meaningful to older people themselves [29] and patient reported outcomes which could be captured in routine data [41, 42]. These efforts would help drive the outputs of big data research into routine practice, as well as drive improvements in data quality and increase awareness of the potential for big data research to underpin service redesign. Engaging public stakeholders also has a role in increasing public awareness of the utility and application of data to improve healthcare, which is critically important for the contract of trust between public and data holders [43].

## Conclusion

Routine data have considerable potential to address knowledge gaps for older people living with multiple conditions and frailty. This includes applied epidemiology and prognostic research, novel clinical trial methods and service evaluations. There are a range of complexities and considerations in ageing routine data research requiring a multidisciplinary approach including clinicians, data scientists, biostatisticians, epidemiologists and trial methodologists. Multidisciplinary collaboration is paramount to harness the potential of routine data to improve delivery of evidence-based care for older people.
